# Host-Parasite Relationships in Porcine Ascariosis: Anticoagulant Potential of the Third Larval Stage of *Ascaris suum* as a Possible Survival Mechanism

**DOI:** 10.3390/ani11030804

**Published:** 2021-03-13

**Authors:** Alicia Diosdado, Fernando Simón, Rodrigo Morchón, Javier González-Miguel

**Affiliations:** 1 Animal and Human Dirofilariosis Group, Laboratory of Parasitology, Faculty of Pharmacy, University of Salamanca, C/Licenciado Méndez Nieto s/n, 37007 Salamanca, Spain; alidm@usal.es (A.D.); fersimon@usal.es (F.S.); rmorgar@usal.es (R.M.); 2 Laboratory of Parasitology, Institute of Natural Resources and Agrobiology of Salamanca (IRNASA-CSIC), C/Cordel de Merinas 40-52, 37008 Salamanca, Spain

**Keywords:** *Ascaris suum*, third-stage larvae, coagulation cascade, anticoagulant, factor Xa, serpin, larval migration, ascariosis, host-parasite relationships

## Abstract

**Simple Summary:**

*Ascaris suum* parasitises pigs all over the world causing a disease responsible for producing reductions in weight gains and damages to several organs of the infected animals that incur huge economic losses for the swine industry. While adult worms of this parasite are located in the small intestine of the host, their larval stages migrate through the bloodstream as an evolutionary advantageous strategy within a hostile environment that confronts host responses such as blood clots formation. The aim of this work is to study the ability of *A. suum* larvae to inhibit blood coagulation as a possible mechanism to control blood clots formation and facilitate their migration. The results showed that these larvae inhibited host blood coagulation and possessed molecules similar to those responsible for inhibiting blood coagulation in pigs. The anticoagulant effect of *A. suum* larvae could constitute a potential survival mechanism for the parasite. Therefore, developing new control strategies directed at this and similar processes could avoid *A. suum* larval migration and the establishment of adult worms in their definitive location, which is necessary to confront the damages and economic losses produced by this parasitosis.

**Abstract:**

In order to evade the response of their hosts, helminth parasites have evolved precise and highly regulated mechanisms, including migration strategies of the larval stages. In regard to porcine ascariosis caused by *Ascaris suum*, its infective third-stage larvae (AsL3) undergo a complex migratory route through the bloodstream of their host before establishing in the small intestine to reach maturation. Despite the benefits attributed to this migration, blood clots formation could compromise larvae survival. The aim of this work was to study the interaction between the cuticle and excretory/secretory antigens of AsL3 and the host coagulation cascade. Larvae were obtained after incubating and hatching *A. suum* eggs, after which the antigenic extracts were produced. Their ability to disrupt the coagulation cascade was studied using anticoagulation and chromogenic assays, and techniques based on electrophoresis. The obtained results showed that both antigenic extracts possessed anticoagulant potential, being able to inhibit the intrinsic, extrinsic and/or common pathways of the blood coagulation cascade as well as the activated factor X. Moreover, three *A. suum* serpin proteins were identified as candidates to inhibit this host coagulation factor. To the best of our knowledge, this study shows, for the first time, the anticoagulant potential of the infective larvae of *A. suum*, which could be used by the parasite as a mechanism to facilitate its invasion and survival in the host.

## 1. Introduction

The beginning of the relationship between pigs and humans dates back thousands of years to when domestication of pigs occurred [1]. Since then, this species has become the main source of meat for human consumption, with the swine industry being the largest producer of meat in the world and an important economic driver in many regions [2,3]. Nevertheless, this industry suffers from significant economic losses, owing to the diseases affecting pigs, among which are soil-transmitted helminthiasis that are easily spread through the faecal-oral route. The most prevalent of these parasites with a worldwide distribution is the causal agent of porcine ascariosis, *Ascaris suum* [4]. Its infective third-stage larvae (AsL3) carry out a complex migratory route through the host tissues before reaching maturation and establishing in the host small intestine. Briefly, AsL3 hatch from ingested eggs in the small intestine and migrate to the caecum and proximal colon to penetrate the mucosa. Then, larvae reach the liver through the hepatic portal system, after which they are carried to the lungs via the systemic circulation. There, AsL3 abandon the circulatory system penetrating the alveolar space to ascend the trachea, reach the oesophagus and return to the small intestine to complete maturation [5]. Once there, adult worms produce growth reduction of infected pigs, but the most severe damages in porcine ascariosis are due to the passage of larvae through the liver and lungs. Both affect animal welfare and are responsible for the huge economic losses, which are directly related to porcine ascariosis [4]. In spite of its importance, optimal diagnostic methods for this parasitosis are not available, and control tools rely on the use of anthelmintic drugs, which entail serious drawbacks that could be improved with the development of vaccination strategies [6]. In order to achieve this purpose, unravelling the molecular basis of the host-parasite relationships in porcine ascariosis is of paramount importance, mainly during the early stages of the infection [7,8].

One of the most striking mechanisms governing host-parasite relationships is the ability of some parasites to manipulate the haemostatic system of their mammalian hosts, including blood coagulation and the fibrinolytic system, which has been related to their success in both the invasion process and their survival inside the host [9,10,11,12]. Blood coagulation is a complex mechanism necessary to stop the haemorrhage when a vascular injury occurs, and includes primary (activation and aggregation of platelets) and secondary (the coagulation cascade) haemostasis. Both mechanisms culminate with the formation of a stable clot between platelets and the cross-linked fibrin deposited over them. Fibrin is the protein responsible for stabilizing the clot and the resultant product of the coagulation cascade, a series of enzymatic chain reactions in which different zymogens (coagulation factors) are activated in their catalytically active serine proteases [13]. The coagulation cascade is divided into two different pathways (intrinsic and extrinsic) that converge at a common point with the activation of factor X (FXa), which participates in thrombin formation, the protein responsible for transforming fibrinogen into fibrin [14]. The regulation of the coagulation cascade depends on the action of several inhibitors, among them antithrombin is the most quantitatively important because it is able to exert its activity against all serine proteases participating in the coagulation process. Antithrombin belongs to the group of serpins (serin protease inhibitors) [15], a noteworthy superfamily of enzymes due to its high evolutionary conservation between mammals and nematodes [16] and its action mechanism based on the formation of a stable complex with its target protein [17].

In a previous study, we have recently demonstrated the capacity of the cuticle and excretory/secretory antigenic extracts of AsL3 (AsL3C and AsL3ES) to activate the host fibrinolytic system, which represents the mechanism of the haemostatic system responsible for dissolving blood clots [18]. In order to complement this study and contribute to the knowledge of the host-parasite relationships in porcine ascariosis, the present work was carried out with the aim of evaluating the ability of AsL3C and AsL3ES to inhibit the coagulation cascade of the host as a potential survival mechanism.

## 2. Materials and Methods

### 2.1. Collection of Third-Stage Larvae of A. suum

AsL3 were obtained, as previously described [19], with minor modifications. Adult female *A. suum* worms were collected from naturally infected pigs from a local abattoir. Parasites were dissected to extract the uterus and both worms and uteri were washed with phosphate-buffered saline (PBS) (0.15 M NaCl, 0.03 M NaH_2_PO_4_H_2_O, 0.08 M Na_2_HPO_4_, pH 7.2). *A. suum* eggs were extracted from the uterus, washed with PBS, suspended in a 2% (*w*/*v*) potassium dichromate (K_2_Cr_2_O_7_) solution and cultured in 24-well plates (Corning, New York, NY, USA) to be incubated at 27 °C for approximately 40 days in a place restricted from light. Once most eggs had embryonated, they were transferred from culture plates to test tubes (Falcon, New Jersey, NJ, USA). The potassium dichromate solution was removed and the eggs were treated with 5‒6% (*w*/*v*) sodium hypochlorite (NaClO) (commercial bleach) at 37 °C for 15 min. After washing at least 5 times with PBS by centrifugation at 200× *g* for 5 min, the eggs were transferred into an Erlenmeyer flask containing glass beads and a magnetic stir bar to be shaken slowly (60 × *rpm*) for approximately 1 h. Once most larvae had hatched, the suspension was poured into a cotton gauze layer (2.5 g) on a Baermann apparatus filled with PBS following the protocol described by Urban et al. [20]. After being cultured at 37 °C in a humidified atmosphere in the presence of 5% CO_2_ overnight, AsL3 were collected from the neck of the funnel and washed with PBS to be subsequently used to obtain the antigenic extracts.

### 2.2. Collection of Antigenic Extracts from AsL3 and Porcine Plasma Samples

AsL3C and AsL3ES antigenic extracts were prepared according to the methodology described by Wedrychowicz et al. [21] and González-Miguel et al. [22], respectively, with minor modifications. To isolate the cuticle antigens, AsL3 were incubated in a 0.25% (*w*/*v*) cetyl trimethyl ammonium bromide (CTAB) solution at 37 °C for 4 h shaking, after which larvae were removed by centrifugation at 200× *g* for 5 min. The supernatant was filtered through a filter of 0.22 μm (Millex, Carrigtwohill, Ireland) and proteins were precipitated with a solution of 0.002 M sodium acetate and 9 volumes of 96% ethanol at −20 °C for 48 h. The suspension was centrifuged at 10,000× *g* for 10 min and the resultant pellet was re-suspended in PBS. In order to obtain de excretory/secretory antigens, the same quantity of AsL3 was cultured in RPMI-1640 medium (Sigma-Aldrich, Saint Louis, MO, USA) supplemented with a 1% (*v*/*v*) antibiotic-antimycotic solution (100×) (Sigma-Aldrich) at 37 °C and 5% CO_2_ for 24 h. After removing larvae by centrifugation at 200× *g* for 5 min, the medium was filtered through a filter of 0.22 μm (Millex). The suspension was dialyzed against water at 4 °C for 48 h shaking and then proteins were concentrated using Amicon Ultra-15 centrifugal filter devices (Millipore, Burlington, VT, USA). Lastly, a cocktail of proteases inhibitors [23] was added to both antigenic extracts. AsL3C and AsL3ES protein concentrations were measured by the BCA protein assay reagent kit (Thermo Fisher Scientific, Waltham, MA, USA) and both antigenic extracts were subsequently stored at −80 °C until their use.

Blood samples were collected from pigs from a local abattoir in tubes containing sodium citrate (0.106 M, 3.8%) (Aquisel, Barcelona, Spain). Samples were immediately centrifuged after collection at 1500× *g* for 15 min to separate plasma from blood cells. Plasma samples were stored at −80 °C until used.

### 2.3. Anticoagulation Assays

The anticoagulant activity of AsL3C and AsL3ES on the host coagulation cascade was assayed following the protocol described by Gan et al. [24] with some modifications. The intrinsic and extrinsic pathways of the coagulation cascade were studied by the activated partial thromboplastin time (APTT) and the prothrombin time (PT) assays, respectively. Both assays also allowed us to study the common pathway of the coagulation cascade. The conversion of fibrinogen into fibrin was evaluated by the thrombin time (TT) assay. The APTT assay was performed by coating multi-well microplates (Corning) with 0.5 μg per well of AsL3C or AsL3ES in the presence of 50 μL of plasma and 50 μL of APTT reagent (BioSystems, Barcelona, Spain). After incubating at 37 °C for 3 min, 50 μL of CaCl_2_ (BioSystems) were added to the previous mixtures to initiate the clotting reaction. For the PT assay, 0.5 μg per well of AsL3C or AsL3ES were incubated with 50 μL of plasma at 37 °C for 2 min. Then, 100 μL of PT reagent (BioSystems) were added to initiate the clotting reaction. Finally, in order to perform the TT assay, 0.5 μg per well of each antigenic extract were incubated with 100 μL of plasma at 37 °C for 2 min, after which the clotting reaction was initiated with 100 μL of TT reagent (BioSystems). Clotting reactions were monitored by measuring the absorbance at 655 nm every 6 s during a period of 42 s in a Microplate Absorbance Reader iMark (Bio-Rad, Hercules, CA, USA). Frozen plasma and reagents were, respectively, thawed and brought to 37 °C just prior to use. PBS was used as a negative control. Each sample was analysed in triplicate.

### 2.4. FXa Inhibition Assay

To evaluate the inhibitory activity of AsL3C and AsL3ES against FXa, a chromogenic assay was performed, as previously described [24], with some modifications. In a final volume of 100 μL, 0.1 μg per well of each antigenic extract were incubated with the native human FXa (Thermo Fisher Scientific) at a final concentration of 4 nM in 4-(2-hydroxyethyl)-1-piperazineethanesulfonic acid (HEPES)-BSA buffer (50 mM HEPES, pH 7.5, 100 mM NaCl, 5 mM CaCl_2_, 1 mg/mL BSA) in multi-well microplates (Corning) at 37 °C for 15 min. The chromogenic substrate S-2765 of FXa (Chromogenix, Bedford, MA, USA) was then added at a final concentration of 800 μM and incubated at 37 °C for 3 h. The hydrolysis of the substrate was monitored by measuring the absorbance at 415 nm every 30 min in a Microplate Absorbance Reader iMark (Bio-Rad). PBS was used as a negative control. Each sample was analysed in triplicate.

### 2.5. Binding of AsL3C and AsL3ES to FXa

In order to determine the likely capacity of AsL3C and AsL3ES proteins to trap FXa into a stable complex, a sodium dodecyl sulfate polyacrylamide gel electrophoresis (SDS-PAGE) was carried out according to the method described by Fonseca et al. [25] with some modifications. Samples of 10 μg of the antigenic extract (AsL3C or AsL3ES), 1 μg of the FXa and a mixture of both compounds (antigenic extract and FXa) were incubated in 5 mM HEPES buffer, pH 7.4, at room temperature for 45 min. All samples were loaded into 10% polyacrylamide gels and protein bands were detected by silver staining after finishing the electrophoresis. Gels were scanned with the GS-800 Densitometer (Bio-Rad). All assays were performed in triplicate.

### 2.6. Mass Spectrometry, Protein Identification and Bioinformatic Analyses

Selected bands containing potential FXa-AsL3 protein complexes were manually excised from the gels and analysed by liquid chromatography and tandem mass spectrometry (LC-MS/MS) at the proteomics facility of the Servei Central de Suport a la Investigació Experimental (SCSIE) of the University of Valencia, Spain. Samples were digested with sequencing grade trypsin (Promega, Madison, WI, USA) (50 ng) [26] and the resultant digestion mixture was dried in a vacuum centrifuge and re-suspended in 7 μL 2% (*v*/*v*) acetonitrile (ACN), 0.1% (*v*/*v*) trifluoroacetic acid (TFA). Five microliters of each sample were loaded onto a trap column (NanoLC Trap, ChromXP, C18-CL 3 μm, 120 Å, 350 μm × 0.5 mm) (Eksigent Technologies, Framingham, MA, USA) and desalted with 0.1% (*v*/*v*) TFA at 3 μL/min during 5 min. Peptides were then loaded onto an analytical column (NanoLC Column, 3 C18-CL-120, 3 μm, 120 Å, 75 μm × 15 cm) (Eksigent Technologies) equilibrated in 5% (*v*/*v*) ACN, 0.1% (*v*/*v*) formic acid (FA). Elution was carried out with a linear gradient of 30% (*v*/*v*) B in A [A: 0.1% (*v*/*v*) FA; B: ACN, 0.1% (*v*/*v*) FA] for 20 min at a flow rate of 300 nL/min. Peptides were analysed in a mass spectrometer nano-ESI-QqTOF (5600 TripleTOF) (AB Sciex, Framingham, MA, USA) and each sample was ionized in a Source Type Optiflow <1 μL Nano applying 3.0 kV to the spray emitter at 200 °C. Analyses were carried out in a data-dependent mode. Survey MS1 scans were acquired from 350–1400 *m*/*z* for 250 ms. The quadrupole resolution was set to LOW for MS2 experiments, which were acquired from 100–1500 *m*/*z* for 25 ms in high sensitivity mode. The following switch criteria were used: charge 2+ to 4+, minimum intensity and 250 counts per second. Up to 100 ions were selected for fragmentation after each survey scan. Dynamic exclusion was set to 15 s. The system sensitivity was controlled by analysing 0.5 μg of K562 trypsin digestion (SCIEX, Framingham, USA). ProteinPilot (SCIEX) default parameters were used to generate peak list directly from 6600 plus TripleTOF files. The Paragon algorithm [27] of ProteinPilot v 5.0 was used to search for in the Swiss-Prot and UniProt_Nematoda databases with the following parameters: trypsin specificity, cys-alkylation, taxonomy non-restricted and search effort set to through with FDR analyses. The protein grouping was done by the Pro group algorithm.

The amino-acid sequences of the interesting proteins for the study resulting from the LC-MS/MS analyses were analysed using the following bioinformatic tools: BLAST searching of the homologous sequences in the NCBI and Swiss-Prot/UniProt databases (http://www.ncbi.nlm.nih.gov/, http://www.uniprot.org/), analysis of conserved protein domains with Prosite (https://prosite.expasy.org/), prediction of signal peptides with SignalP-5.0 [28] (http://www.cbs.dtu.dk/services/SignalP) and multiple sequence alignment with Clustal Omega (https://www.ebi.ac.uk/Tools/msa/clustalo/) (accessed on 4 November 2020 for all websites).

### 2.7. Statistical Analysis

Anticoagulation assays and FXa inhibition assay results were analysed with the Student’s *t*-test. Data were expressed as the mean ± standard deviation (SD) of three independent experiments. Significant differences were defined as a *p*-value of <0.05 for a confidence level of 95%.

## 3. Results

### 3.1. AsL3C and AsL3ES Possess Anticoagulant Activity

**Figure 1:**
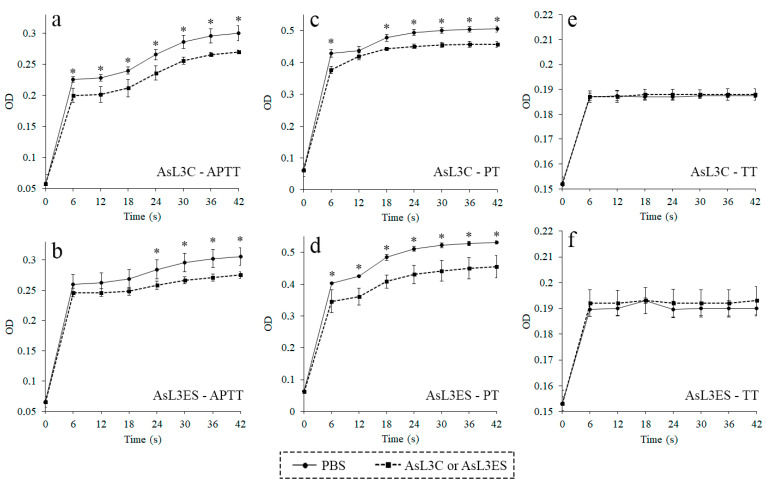
Anticoagulant activity of AsL3C (**a**,**c**,**e**) and AsL3ES (**b**,**d**,**f**) evaluated by measuring the APTT (**a**,**b**), the PT (**c**,**d**) and the TT (**e**,**f**). Plasma from pigs was incubated with 0.5 μg of the antigenic extract (■) or with PBS as a negative control (●), and the corresponding reagent (APTT, PT or TT). Each point represents the mean of three replicates ± SD. Significant differences (*p* < 0.05) are marked with an asterisk (*).

The anticoagulant activity of AsL3C and AsL3ES was evaluated by the anticoagulation assays APTT, PT and TT. The results of APTT and PT assays showed that both antigenic extracts possessed anticoagulant activity, since their optical densities were significantly lower than those obtained for the negative controls over time (*p* < 0.05). No significant differences were found between groups for both antigenic extracts in the TT assay (*p* < 0.05) (Figure 1).

### 3.2. AsL3C and AsL3ES Inhibit FXa

**Figure 2:**
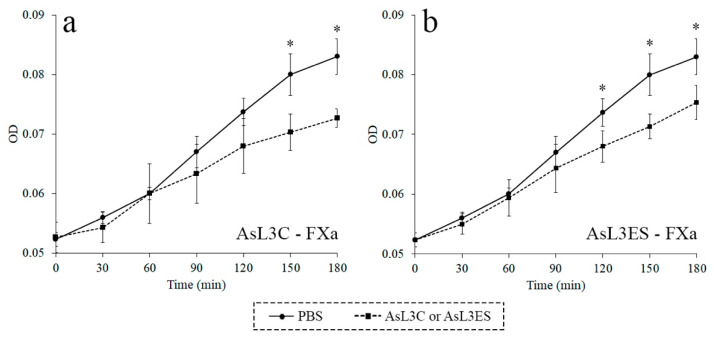
Inhibition of FXa by AsL3C (**a**) and AsL3ES (**b**). 0.1 μg of the antigenic extract (■) were incubated with 4 nM FXa and 800 μM S-2765 in a total volume of 100 μL. The presence of the antigenic extract was replaced by PBS as a negative control in the previous reaction mixtures (●). Each point represents the mean of three replicates ± SD. Significant differences (*p* < 0.05) are marked with an asterisk (*).

The inhibitory activity of AsL3C and AsL3ES against FXa was studied by a chromogenic assay. Optical densities were significantly lower for AsL3C and AsL3ES extracts than those obtained for the negative controls, which showed the inhibitory properties of both antigenic extracts against FXa over time (*p* < 0.05) (Figure 2).

### 3.3. AsL3C and AsL3ES Bind FXa

**Figure 3:**
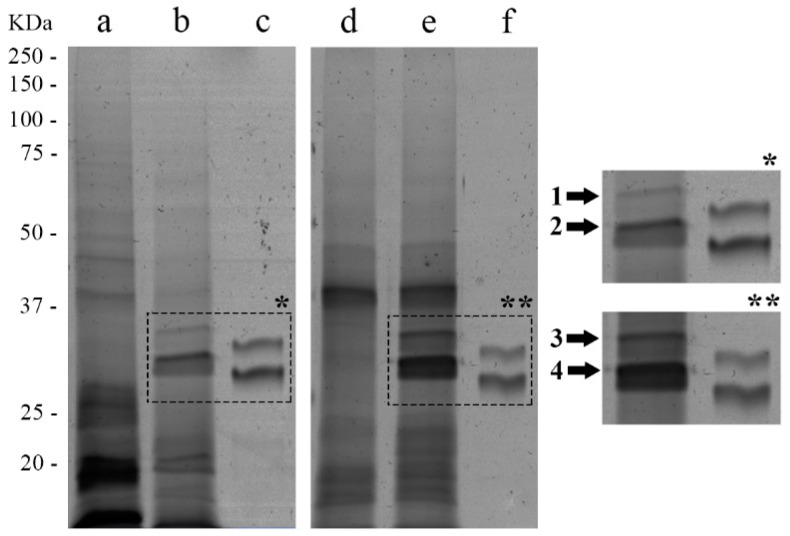
Binding of AsL3C (**a**–**c**) and AsL3ES (**d**–**f**) to FXa by SDS-PAGE. 10 μg of the antigenic extract (**a**,**d**), 10 μg of the antigenic extract + 1 μg of the FXa (**b,e**) and 1 μg of the FXa (**c**,**f**) were pre-incubated in 5 mM HEPES buffer, pH 7.4. The reference of molecular weights is indicated on the left. The bands marked with arrows correspond to the FXa in the samples incubated with AsL3C (*) and AsL3ES (**).

To evaluate whether AsL3C and AsL3ES were able to trap FXa into a stable complex, a SDS-PAGE was performed. The electrophoresis gel revealed that both antigenic extracts bound FXa since two bands in the lanes containing the pre-incubated mixture of AsL3C/AsL3ES and FXa appeared in a larger molecular weight (~31 and ~34 kDa) than in the lanes containing only the coagulation factor, in which they were respectively located at ~29 and ~32 kDa. These bands were absent in the lanes containing only the antigenic extracts (Figure 3). In addition, other changes were observed in the electrophoresis gel. In the case of AsL3C, bands between ~23 and ~28 kDa and those located under 20 kDa in the sample prepared only in the presence of the antigenic extract were, respectively, absent and less stained in the pre-incubated mixture. With respect to AsL3ES, two bands (at 21.9 and 23.1 kDa) located in the pre-incubated mixture were, respectively, absent and less stained in the sample prepared only in the presence of the antigenic extract (Figure 3).

### 3.4. Identification of Potential Inhibitors of FXa in AsL3C and AsL3ES

The two bands containing potential FXa-AsL3 protein complexes in the pre-incubated mixtures of the coagulation factor with each antigenic extract (Figure 3) were manually excised from the silver stained SDS-PAGE gels and identified by LC-MS/MS. Among different parasite proteins identified in the analysed bands, three serpins deposited in databases as *A. suum* proteins were found (Table 1). One of these proteins appeared in AsL3C, another protein derived from AsL3ES and the last one was identified in both extracts. These candidates were identified in the band located in the highest molecular weight in both antigenic extracts. The bands located in the lowest molecular weight did not show any serpin. *Homo sapiens* coagulation factor X was found in all analysed bands. All these proteins were identified with a confidence percentage of 99% according to the equation [ProtScore = −log(1 − (percent confidence/100))] in the ProteinPilot. The bioinformatic analyses of the deduced amino-acid sequences of the selected proteins allowed us to identify the cleavage site of the signal peptide and the serpin signature sequence in one and two proteins, respectively. These data, together with the UniProt accession code of the proteins, their description, theoretical molecular weights (MW) and length of the amino-acid sequences are shown in Table 1.

**Figure 4:**
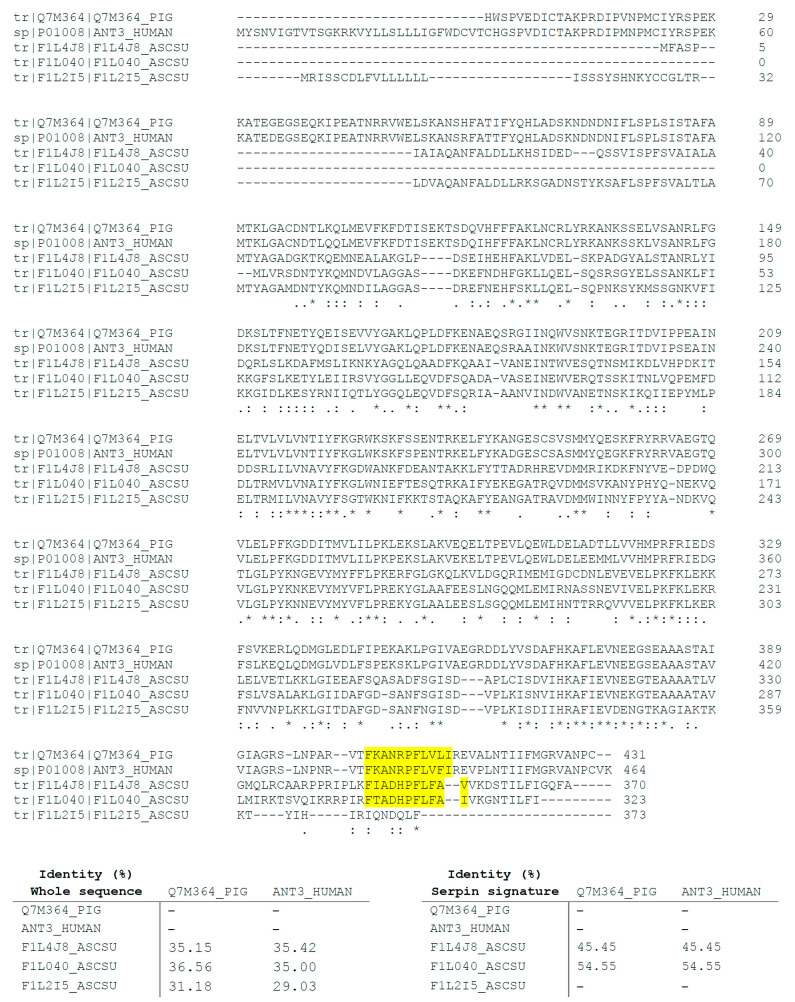
Alignment of the AsL3C and AsL3ES serpins (F1L4J8_ASCSU, F1L040_ASCSU and F1L2I5_ASCSU) identified by LC-MS/MS with the antithrombin III from *S. scrofa domesticus* (Q7M364_PIG) and *H. sapiens* (ANT3_HUMAN). The amino-acids conserved in all sequences are labelled with asterisks while the conservative and semiconservative substitutions are respectively labelled with two and one point. The identity percentage of both the whole sequences and the serpin signature domains between AsL3 serpins and pig and human antithrombin III is indicated in the tables below. The serpin signature sequences are highlighted in yellow.

The three identified serpins were also compared by multiple sequence alignment to the physiological inhibitor of FXa in mammals, the antithrombin III from *Sus scrofa domesticus* and *H. sapiens* (accession codes to UniProt: Q7M364_PIG and ANT3_HUMAN, respectively). The results of this analysis revealed identity percentages that ranged between 31.18% and 36.56% in the case of *S. scrofa domesticus* and 29.03% and 35.42% for *H. sapiens*. This analysis was also carried out to compare sequence alignment between the serpin signature domains of F1L4J8_ASCSU and F1L040_ASCSU and the physiological inhibitors of FXa in mammals. The results showed identity percentages of 45.45% and 54.55% between serpin signature domains of serpins identified in AsL3C and AsL3ES extracts and those domains of antithrombin III from mammals, respectively (Figure 4).

## 4. Discussion

Host-parasite relationships have evolved as a successful mechanism, allowing parasites to confront and evade the response of their hosts as well as to facilitate their adaptation to the host tissues. These strategies of manipulation need to be properly regulated, especially in some tissues such as vascular locations, where blood parasites are exposed to multiple aggression mechanisms from the host, among these are the generation of thromboembolisms [12,29]. In this context, the ability of some parasites to alter the host coagulation cascade has been widely studied [30,31]. While *A. suum* is considered to be an intestinal parasite due to its definitive location, its infective L3 stage undergoes an extensive migration through the host bloodstream that begins and ends in the same location [5]. In this regard, tissue migration by parasitic nematode larvae has been proposed as a selectively advantageous strategy facilitating their establishment in the host [32]. For this reason, the aim of this work was to study the ability of AsL3 to interact with the host coagulation cascade as a potential survival mechanism through the use of parasite extracts containing cuticle and excretory/secretory proteins representing the host-parasite interface.

The results obtained in the present study revealed that AsL3C and AsL3ES inhibited the intrinsic, extrinsic and/or common pathways of the coagulation cascade before the transformation of fibrinogen into fibrin, since the APTT and PT assays were significantly altered in the presence of both antigenic extracts, but no significant change was found in the TT assay. These results correlate with those obtained in other studies using similar methodologies for both helminth and arthropod parasites [33,34,35,36,37] as well as reinforce the studies developed with the adult worms of *A. suum* and its closely related species *A. lumbricoides* during the 1980s and 1990s [38,39].

Specifically, the disruption of the coagulation cascade by parasites has been demonstrated to be influenced by the inhibitory activity of parasite antigens on the host coagulation factors [40,41,42,43]. In this sense, our data showed that both antigenic extracts, AsL3C and AsL3ES, possessed inhibitory activity against FXa. Due to its pivotal role in thrombin formation, FXa is one of the most studied coagulation factors in host-parasite relationships, being the main target of numerous anticoagulant molecules identified in parasites [24,37,44,45,46,47]. Furthermore, FXa has been proposed as a promising target for the development of new anticoagulant agents [48], among which a number of anticoagulant peptides from some species of ticks and hookworms have already been assayed [49].

Antithrombin, the main physiological inhibitor of FXa and other coagulation factors in mammals, belongs to the group of serpins, and thus parasite serpins have been widely suggested to be responsible for regulating the coagulation cascade of their hosts [9,16,50,51,52,53]. The inhibitory mechanism of serpins entails the formation of an irreversible complex between these enzymes and their target proteins, hence this complex can be studied by SDS-PAGE [17]. According to this, our results revealed the formation of potential inhibitory complexes between each of the studied antigenic extracts (AsL3C/AsL3ES) and FXa, which correlates with other similar studies [37,54]. The subsequent analysis by LC-MS/MS of these complexes allowed us to identify three *A. suum* serpins as potential candidates to inhibit FXa. It is worth mentioning that, in addition to their role in disrupting coagulation cascade, helminth serpins have also been related to other important survival mechanisms such as the modulation of inflammation and the host immune system [55,56,57]. In regards to porcine ascariosis, complexes formed between *A. suum* serpins and host proteases have been postulated as a mechanism to mask the larvae surface in order to evade the host immune response during their migration [58]. In addition, serpins have also been related to *A. suum* protection from digestive degradation by the host proteolytic enzymes [58]. Despite their important role in parasite survival, some data suggest that serpins released by parasites could be detected by the host immune system because of their high immunogenic potential, so they have been proposed as interesting targets for the development of novel vaccination strategies [16]. Finally, the multiple sequence alignment carried out to compare AsL3C and AsL3ES identified serpins with pig and human antithrombin showed low homology between nematode and mammalian serpins. However, the similarity was notably higher when the serpin signature sequences were compared separately. These results are in accordance with those obtained for other nematode parasites [37] and the statement postulated by Zang and Maizels [16] that suggests that nematode and mammalian serpins have low overall homology, but they are highly conserved at most of the key amino-acid residues necessary to maintain the structure and function of the protein such as the serpin signature.

The fact that different parasites infecting different host species develop similar strategies to regulate the coagulation cascade of their hosts denotes their importance and evolutionary conservation. For AsL3, blood clots could constitute a barrier to migrate through the host bloodstream and tissues, and moreover, to establish in the host since the activation of coagulation is involved in the immune response during infections [59]. The anticoagulant potential of AsL3 could limit thrombin production and consequently fibrin generation, avoiding blood clots formation. This mechanism could occur in the AsL3 immediate intravascular habitat or at a systemic level, since similar results were obtained in this study by both their cuticle and excretory/secretory products. Therefore, this strategy could help the parasite to migrate, evade the host immune system and survive, as it has been postulated for other nematode and trematode parasites [31,35,37,60]. In addition, coagulation inhibition by AsL3 could contribute to aggravate the pathological processes produced in the infected pigs. In this sense, this phenomenon could be responsible for producing bleedings and haemorrhagic lesions that emerge in the intestinal mucosa, liver and lungs as a result of larvae penetration and migration [4,8,61,62]. These lesions not only affect pig welfare, but they also cause important economic losses for the swine industry due to condemnation or downgrading of damaged organs [4].

All these results are in line with those obtained in the previous study, which showed the pro-fibrinolytic potential of AsL3 [18]. The present work reinforces the idea that AsL3 could modulate the haemostatic system of their host for their own benefit, not only through the dissolution of blood clots, but also preventing their formation. In addition, the results obtained in the present study could contribute to expand the knowledge on the host-parasite relationships in human ascariosis caused by *A. lumbricoides*, since this species has a similar life-cycle to *A. suum* [5] and growing studies are pointing out that hybridisation and cross-transmission between both species occur [63,64,65,66].

## 5. Conclusions

To our knowledge, we demonstrate for the first time the ability of AsL3 to inhibit the coagulation cascade, at least at the FXa level, by the anticoagulant activity of both their cuticle and excretory/secretory antigenic extracts, as well as identified AsL3 serpins as potential inhibitors of FXa. The inhibition of blood clots formation by the parasite could be an important mechanism to migrate, establish and survive in the host. The diverse strategies of AsL3 to interact with the haemostatic system of the pig suggest the importance of the regulation of blood clots formation for the parasite and the complexity of host-parasite relationships in porcine ascariosis. Thus, knowing these relationships in the early larval stage is of paramount importance to found new control strategies such as vaccination. Future studies could identify and characterise these anticoagulant molecules, which potentially constitute new targets to the control of ascariosis, and moreover, a source of new antithrombotic agents for human therapy.

## Figures and Tables

**Table 1:** Serpins of AsL3C and AsL3ES identified by LC-MS/MS from the analysed bands. All identifications belonged to *A. suum* proteins deposited in databases. The band numbers correspond to those numbers indicated in Figure 3. Amino-acid, aa.

Band Number	Antigenic Extract	Accession Code	Description	Theoretical MW (kDa)	Length (aa)	Cleavage Site (aa)	Serpin Signature (aa)
1	AsL3C	F1L4J8_ASCSU	Serpin B6	41.4	370	‒	FIADHPFLFAV (347‒357)
3	AsL3ES	F1L040_ASCSU	Serpin-like protein	36.7	323	‒	FTADHPFLFAI (304‒314)
1, 3	AsL3C, AsL3ES	F1L2I5_ASCSU	Serpin B6	42.3	373	22‒23	‒

## Data Availability

Not Applicable.
